# The POSEIDON-R compartment model for the prediction of transport and fate of radionuclides in the marine environment

**DOI:** 10.1016/j.mex.2018.10.002

**Published:** 2018-10-06

**Authors:** V. Maderich, R. Bezhenar, Y. Tateda, M. Aoyama, D. Tsumune, K.T. Jung, G. de With

**Affiliations:** a Institute of Mathematical Machine and System Problems, Kiev, Ukraine; b Environmental Science Research Laboratory, Central Research Institute of Electric Power Industry, Chiba, Japan; c Institute of Environmental Radioactivity, Fukushima University, Fukushima, Japan; d Korea Institute of Ocean Science and Technology, Busan Metropolitan City, Republic of Korea; e Nuclear Research and Consultancy Group, Arnhem, Netherlands

**Keywords:** Compartment model for the prediction of transport and fate of radionuclides in the marine environment, Box modelling, Dynamic food chain model, Radioactivity in the marine environment

## Abstract

A detailed description of the advanced version of compartment model POSEIDON-R for the prediction of transport and fate of radionuclides in the marine environment is given. The equations of transfer of radionuclides in the water and bottom sediment compartments along with the dynamical food chain model are presented together with dose module to assess individual and collective doses to the population due to the regular and accidental releases of radionuclides. The method for the numerical solution of model equations is also presented. The modelling results for the northeast Atlantic shelf seas were compared with measurements of ^137^Cs.

• The three-dimensional compartment model POSEIDON-R describes the transfer of radionuclides and their daughter products in marine environment as a results of regular or accidental releases. This includes any transfer through the water column and sediments.
• The model is complemented by a dynamic food chain model for transfer of radioactivity in pelagic and benthic food webs.
• The dose module in the model calculates internal and external doses for humans and non-human biota.

## Graphical abstract

**Figure:**
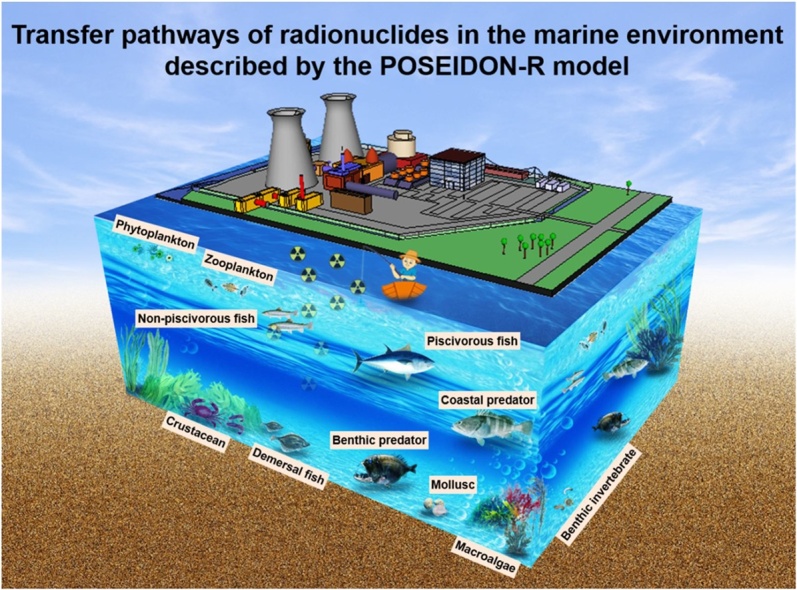

**Specifications Table**

**Table:** 

**Subject Area**	*Environmental Science*
**More specific subject area:**	*Radioactivity transfer in marine environment*
**Method name:**	Compartment model for the prediction of transport and fate of radionuclides in the marine environment
**Name and reference of original method**	Lepicard S., Raffestin D., Rancillac F., 1998. POSEIDON: A dispersion computer code for assessing radiological impacts in a European sea water environment. Radiation Protection Dosimetry 75, (1-4), 79–83. Lepicard S., Heling R., Maderich V., 2004. POSEIDON-R/RODOS models for radiological assessment of marine environment after accidental releases: application to coastal areas of the Baltic, Black and North Seas. J. Environ. Radioactivity 72, 153-161.
**Resource availability**	

## Method details

Despite the rapid development of numerical hydrodynamic models, the compartment (box) models continue to play an important role in the modelling of transport and fate of radioactivity in marine environments because of its relative simplicity and flexibility [1]. Originally, POSEIDON model [2] was developed to assess the radiological consequences of instantaneous or continuous releases of a mixture of radionuclides in European sea waters. For this purpose a box modelling approach as described in [3] was adopted. The model calculated the radioactivity transfer in the water, bottom sediments and biota to estimate doses for humans resulting from the consumption of contaminated seafood. The modified model became a part of European Decision Support System for emergency response to nuclear accidents RODOS [4,5] under the name POSEIDON-R. In this model a transfer of radionuclides to marine organisms was described by means of a dynamical uptake model BURN [6], taking into account the trophic level of the organisms. Simultaneously, the list of sources of radionuclides due the accidental releases was also essentially extended.

In the paper we describe an advanced version of POSEIDON-R that is supplemented with a new biological uptake model describing migration of activity through the pelagic and benthic food webs [7] and modified dose modules with extended pathways for human exposure from marine releases of radioactivity. The model was applied to the northeast Atlantic shelf seas and the modelling results presented in this work were compared with measurements.

### Dispersion of activity in water and sediments

**Fig. 1:**
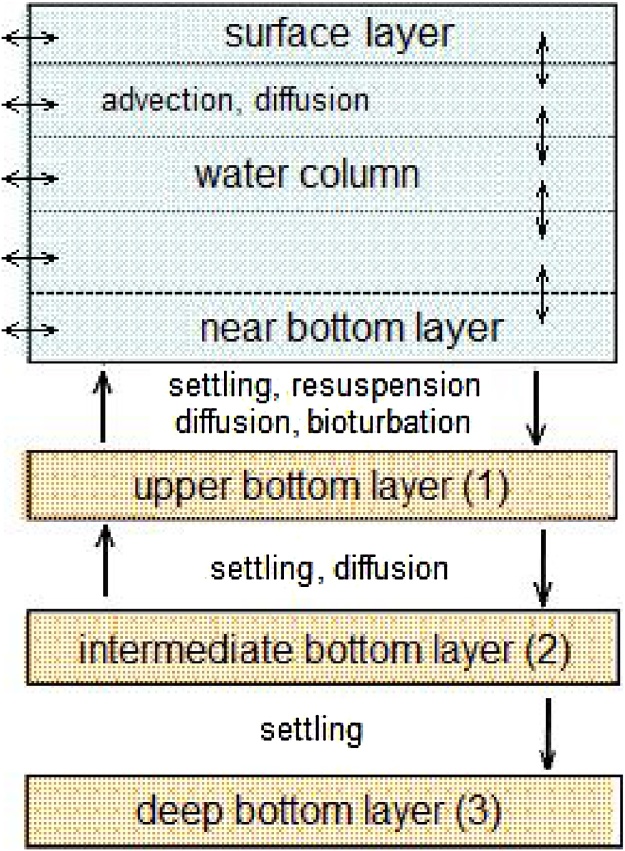
Vertical structure and radionuclide transfer processes in the compartment model POSEIDON-R.

The POSEIDON-R 3D compartment model simulates transfer of radioactivity in the water column and bottom sediment (Fig. 1). The water column compartment is vertically subdivided into layers. The suspended matter is settling in the water column. The radionuclide concentration in the water compartment is governed by a set of differential equations that describe i. the temporal variation in the concentration, ii. the exchange of radionuclides between adjacent compartments and between radionuclides in suspension and in the bottom sediment, iii. and radioactivity sources and decay. Exchanges among the water column layers are described by radionuclide fluxes due to advection, sediment settling, and turbulent diffusion. The bottom sediments are divided into three layers, and the transfer of radioactivity between the upper sediment layer and the water column resulting from resuspension, diffusion and bioturbation, and between the upper and middle sediment layers, resulting from diffusion only, are described as shown in Fig. 1. Downward burial processes operate in all three sediment layers.

The POSEIDON-R equations are obtained by averaging the three dimensional transport equations (see [8]) for the dissolved radionuclide concentration in the water column and the concentration in three layers of the bottom sediment assuming that partitioning between dissolved and particulate fractions is described by a distribution coefficient *K*_d_ (m^3^ t^−1^). The equations for the water column layers, upper and middle sediment layer read as follows:

(1) $$\frac{\partial C_{0ik}}{\partial t}=\sum_{m}\sum_{j}\left[\frac{F_{jimk}}{V_{0ik}}C_{0jm}-\frac{F_{ijkm}}{V_{0jm}}C_{0ik}\right]+\gamma_{0ik}C_{0(i+1)k}-(\gamma_{1ik}+\lambda )C_{0ik}+\frac{L_{t,k}}{h_{1k}}\gamma_{2k}C_{1k}+Q_{sik},$$

(2) $$\frac{\partial C_{1k}}{\partial t}=-(\gamma_{2k}+\gamma_{3k}+\lambda )C_{1k}+\frac{h_{1k}}{L_{t,}k}\gamma_{1k}C_{01k}+\frac{L_{m,k}}{L_{t,k}}\gamma_{4k}C_{2k},$$

(3) $$\frac{\partial C_{2k}}{\partial t}=-(\gamma_{4k}+\gamma_{5k}+\lambda )C_{2k}+\frac{L_{t,k}}{L_{m,k}}\gamma_{3k}C_{1k}.$$

Here *C*_0_*_ik_* is the spatially averaged concentration of radionuclide (Bq m^−3^) in the water column layer *i* of box *k*; *i* = 1 corresponds to the near bottom water column layer; *C*_1_*_k_* and *C*_2_*_k_* are the averaged concentration of radionuclide (Bq m^−3^) in the upper and middle sediment layers of box *k*, respectively; *λ* (y^-1^) is the radionuclide decay constant; *F_ijkm_* is the water flux (t y^-1^) from layer *i* of box *k* to layer *j* of box *m*; *V*_0_*_ik_* is the volume (m^3^) of layer *i* of box *k*; *h_ik_* is the thickness (m) of the water column layer *i* of box *k* ; *L_t,k_* and *L_m,k_* are the thicknesses (m) of top and middle bottom sediment layers of box *k*, respectively; *Q_sik_* is the source of the activity (Bq y^-1^) in layer *i* of box *k*; *γ*_0_*_ik_*…*γ_5k_* are the transfer rates: *γ*_0_*_ik_* is a transfer rate of radioactivity due to sinking of the suspended sediments from the upper layer in the water column in box *k*, *γ*_1_*_ik_* is a transfer rate of radioactivity due to sinking of the suspended sediments to the lower layer in the water column or to the top sediment layer in box *k*, *γ*_2_*_k_* is a transfer rate of radioactivity from the top sediment layer to the near bottom layer in the water column in box *k*, *γ*_3_*_k_* is a transfer rate of radioactivity from the top to middle sediment layer in box *k*, *γ*_4_*_k_* is a transfer rate of radioactivity from the middle to top sediment layer in box *k*, *γ*_5_*_k_* is a transfer rate of radioactivity from the middle to deep sediment layer in box *k*. For the surface water layer, these coefficients are as follows:

(4) $$\gamma_{0ik}=0,$$

(5) $$\gamma_{1ik}=\frac{K_{d}SSW_{k}}{h_{ik}(1+K_{d}SS_{k})},$$

(6) $$\gamma_{2k}=0.$$

For the layers in the water column below the surface water layer, the coefficients are defined as:

(7) $$\gamma_{0ik}=\frac{K_{d}SSW_{k}}{h_{ik}(1+K_{d}SS_{k})},$$

(8) $$\gamma_{1ik}=\frac{K_{d}SSW_{k}}{h_{ik}(1+K_{d}SS_{k})},$$

(9) $$\gamma_{2}k=0.$$

In the near bottom layer located in the water column just above the bottom sediment, the coefficients are defined as:

(10) $$\gamma_{0ik}=\frac{K_{d}SSW_{k}}{h_{ik}(1+K_{d}SS_{k})},$$

(11) $$\gamma_{1ik}=\frac{1}{\left(1+K_{d}SS_{k}\right)}\left[\frac{K_{d}SSW_{k}}{h_{ik}}+\frac{D}{L_{b,k}\min(L_{b,k},L_{t,k})}+\frac{K_{d}SSW_{k}B_{k}}{L_{b,k}\min(L_{b,k},L_{t,k})}\right],$$

(12) $$\gamma_{2k}=\frac{1}{R_{k}}\frac{D}{L_{t,k}\min(L_{b,k},L_{t,k})}+\frac{\left(R_{k}-1\right)}{R_{k}}\frac{B_{k}}{L_{t,k}\min(L_{b,k},L_{t,k})}+\beta_{0}\frac{\left|V_{k}\right|}{L_{t,k}},$$

(13) $$\gamma_{3k}=\frac{R_{k}-1}{R_{k}}\frac{SSW_{k}}{L_{t,k}(1-\varepsilon_{k})\rho_{k}}+\frac{1}{R_{k}}\frac{D}{L_{t,k}\min(L_{t,k},L_{m,k})},$$

(14) $$\gamma_{4}=\frac{1}{R}\frac{D}{L_{m,k}\min(L_{t,k},L_{m,k})},$$

(15) $$\gamma_{5k}=\frac{\left(R_{k}-1\right)}{R_{k}}\frac{SSW_{k}}{L_{m,k}(1-\varepsilon_{k})\rho_{k}}.$$

Here *L_b,k_* is the thickness (m) of the bottom boundary layer in the water column of box *k*, *SS_k_* is the concentration of suspended sediments (t m^−3^) in box *k*, obtained from observations or model simulation, *W_gk_* is the settling velocity (m y^−1^) of suspended sediments in box *k* calculated as function of particles size; *SSW_k_*=*SS_k_W_gk_* is the sedimentation flux (t m^−2^ y^−1^) in box *k*; *D* is the coefficient of vertical diffusion (m^2^ y^−1^) in the bottom layers; *B_k_* is the coefficient of bioturbation (m^2^ y^−1^) in the top sediment layer of box *k*; *ε_k_* is the porosity of the bottom sediment in box *k*; *ρ_k_* is the density of sediment particles (t m^−3^) in box *k*; and the coefficient *R_k_* is defined as

(16) $$R_{k}=1+\frac{\rho_{k}(1-\varepsilon_{k})}{\varepsilon_{k}}K_{d}.$$

The mass concentration of radioactivity in dry sediment for top and middle sediment layers ${\tilde{C}}_{1k}$ and ${\tilde{C}}_{2k}$ (Bq kg^−1^), respectively, is calculated as

(17) $$\begin{array}{cc} {\tilde{C}}_{1k}=\frac{C_{1k}}{\rho_{k}\left(1-\varepsilon_{k}\right)}; & {\tilde{C}}_{2k}=\frac{C_{2k}}{\rho_{k}(1-\varepsilon_{k})} \end{array}.$$

It was found [7] that the standard parameterization of exchange between water column and bottom sediments, which includes sorption/desorption reactions, molecular diffusion and bioturbation, downplays fluxes of activity between water column and bottom sediments in shallow areas with energetic currents. The same difficulties occurred for the Irish and North Seas with strong tidal currents. Therefore, the model was complemented by a parameterization of resuspension mechanism allowing more accurate description of the upper bottom sediments self-cleaning. The resuspension was parameterized as an additional flux directed from the upper sediment layer to the water (last term in the Eq. (12)), which is proportional to the near-bottom velocity in shallow boxes. Parameter *β*_0_ was estimated as $2\cdot 10^{-8}$ and $4\cdot 10^{-8}$ for seas without and with strong tidal currents, respectively, |*V_k_*| is the velocity module (m yr^−1^) in near bottom water layer of box *k* defined as a sum of all fluxes of water between the near bottom water layer of box *k* and neighbouring water layers *F_kn_* divided by areas of faces between these layers *S_kn_*

(18) $$\left|V_{k}\right|=\sum_{n}F_{kn}/\sum_{n}S_{kn}$$

Equations for the daughter products are similar to Eqs. (1), (2), (3) [2]. The differences between those for the parent and daughter radionuclides are (a) new decay constant $\lambda_{d}$ specific for the daughter radionuclide and (b) additional source terms in the RHS of (1)-(3) representing input from the parent to the daughter radionuclide

### Dynamic food web model

**Fig. 2:**
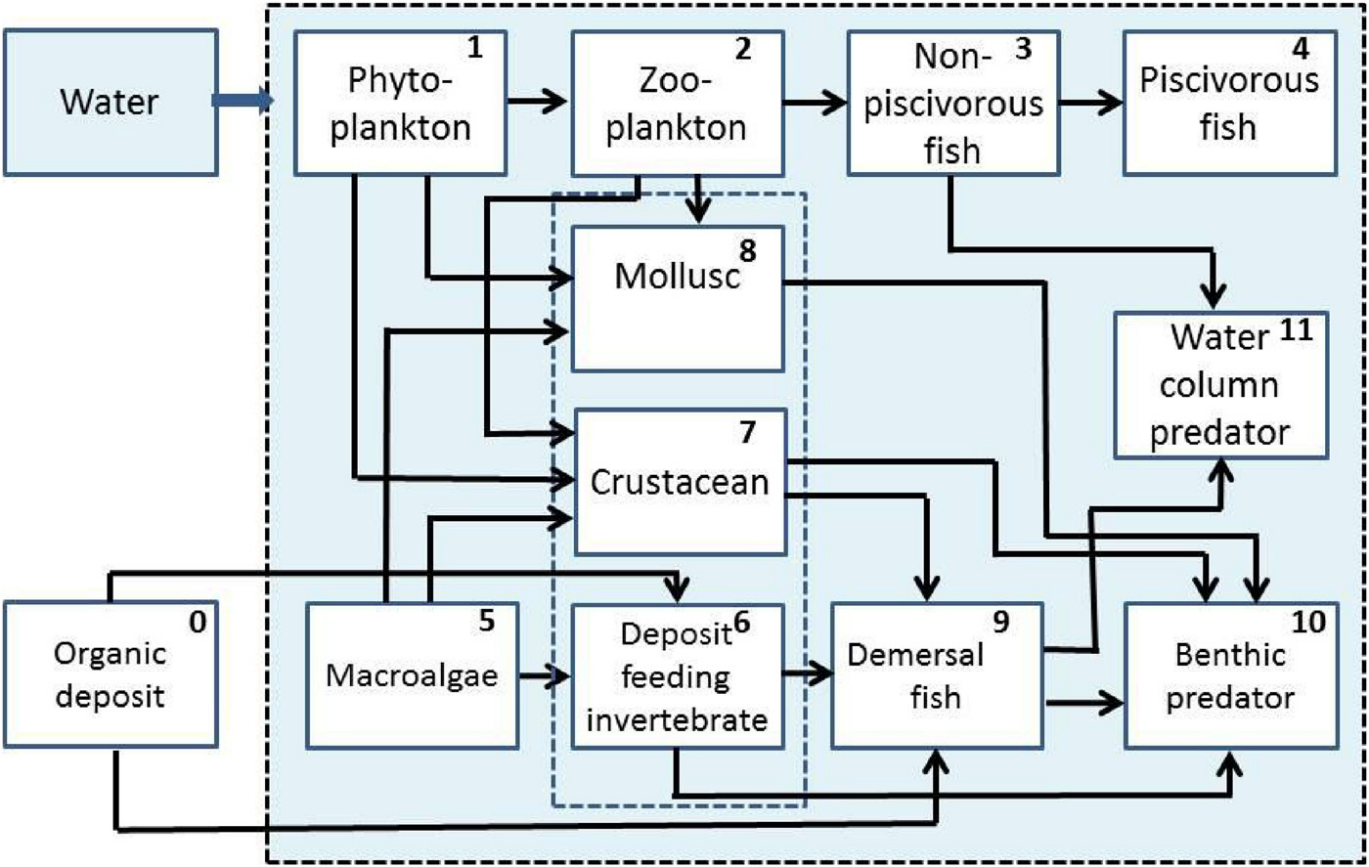
Radionuclide transfer from the water and bottom sediment boxes to marine organisms [7]. The radionuclide transfers among marine food web compartments are given for 11 types of marine organisms.

The recent biota model intercomparison [9] shows that dynamic biota models, which handle situations out from equilibrium, perform better than equilibrium models for the radioecological dose assessment after nuclear accidents. The biota model in POSEIDON-R is a dynamic food web model based on the approach developed by Heling et al. [6]. In the food web model, marine organisms are grouped into classes according to trophic level and species type (Fig. 2). Radionuclides are also grouped into classes according to the fish tissue type in which they are preferentially accumulated (e.g., ^137^Cs tends to accumulate in muscle). These simplifications allow for a limited number of standard input parameters. The scheme of transfer of radionuclides through the marine food web is shown in Fig. 2. The different food chains exist in the pelagic zone and in the benthic zone. Pelagic organisms are grouped into a primary producer (phytoplankton) and consumers: zooplankton, forage (non-piscivorous) fish and piscivorous fish. The pelagic food web was implemented in the compartmental POSEIDON-R model [4,10,11]. The benthic food web includes three primary pathways for radionuclides: (i) transfer from water to macroalgae, then to grazing invertebrates; (ii) transfer through the vertical flux of detritus and zooplankton faces to detritus-feeding invertebrates; and (iii) transfer through contaminated bottom sediments to deposit-feeding invertebrates. External boxes in Fig. 2 show the concentrations of radionuclides in the water and in the organic deposit, which is in instantaneous equilibrium with the upper layer of the bottom sediment, calculated by the above described POSEIDON-R model. In the benthic food chain the radioactivity is transferred from the deposit feeding invertebrates to the demersal fish, and to the bottom predators. The components of this system are crustaceans (e.g detritus-feeders), molluscs (filter-feeders) and coastal predators feeding in the whole water column of shallow coastal waters [7]. Along with the food web, all organisms take radionuclides directly from water.

Due to the rapid uptake from water and the short retention time of radioactivity, the concentration of radionuclides in phytoplankton is calculated using the Biological Concentration Factor (*BCF*) approach [12]. For the macroalgae, a dynamic model is used to describe radionuclide concentrations due to the longer retention times

(19) $$\frac{dC_{ma}}{dt}=\left(CF_{ma}C_{w}-C_{ma}\right)\frac{\ln2}{T_{0.5,ma}},$$

where *C_w_* and *C_ma_* are the radionuclide concentration in the water and macroalgae, respectively, *CF_ma_* is the corresponding *BCF*, *T*_0.5,_*_ma_* is the biological half-life of the radionuclide in the macroalgae, *t* is the time. The concentration of a given radionuclide in other considered marine organisms is described by the following differential equation:

(20) $$\frac{dC_{i}}{dt}=a_{i}K_{f,i}C_{f,i}+b_{i}K_{w,i}C_{w}-\frac{\ln2}{T_{0.5,i}}C_{i},$$

where *C_i_* and *C_f,i_* are the radionuclide concentration in the *i*-th marine organisms and their food, respectively, *a_i_* is the food extraction coefficient (assimilation rate), *b_i_* is the water extraction coefficient, *K_f,i_* is the food uptake rate, *K_w,i_* is the water uptake rate and *T*_0.5,_*_i_* is the biological half-life of the radionuclide in the organism.

The activity concentration in the food of a predator *C_f_* is expressed by the following equation, summing for a total of *n* prey types,

(21) $$C_{f}=\sum_{i=0}^{n}C_{prey,i}\;P_{prey,i}\;\frac{{drw}_{pred}}{{drw}_{prey,i}},$$

where *C_prey,i_* is the activity concentration in prey of type *i*, *P_prey,i_* is preference for prey of type *i*, *drw_pred_* is the dry weight fraction of predator, and *drw_prey_* is the dry weight fraction of prey of type *i*. The index “0″ corresponds to the organic deposit in bottom sediments. Values of the model parameters are discussed in [7] and are given in Table 1, Table 2, Table 3.

**Table 1:** Parameters of dynamical food chain model.

*i*	Organism	Parameters^a^					
		drw	*K*_1_ (d^−1^)	*a*	*K*_w_ (m^3^(kg d)^−1^)	*b*	*T*_0.5_ (d)
1	Phytoplankton	0.1	–	–	–	–	–
2	Zooplankton	0.1	1.0	0.2	1.5	0.001	5
3	Non-piscivorous fish	0.25	0.03	0.5	0.1	0.001	Table 3
4	Piscivorous fish	0.3	0.007	0.7	0.075	0.001	Table 3
5	Macroalgae	0.1	–	–	0.6	0.001	60
6	Deposit feeding invertebrates	0.1	0.02	0.3	0.1	0.001	15
7	Molluscs	0.1	0.06	0.5	0.15	0.001	50
8	Crustaceans	0.1	0.015	0.5	0.1	0.001	100
9	Demersal fish	0.25	0.007	0.5	0.05	0.001	Table 3
10	Bottom predator	0.3	0.007	0.7	0.05	0.001	Table 3
11	Coastal predator	0.3	0.007	0.7	0.075	0.001	Table 3

a See text for definitions of parameters.

**Table 2:** Food preference for prey of type *i*, for prey of type *j*.

Predator Prey	2	3	4	6	7	8	9	10	11
0				0.5			0.1		
1	1.0				0.6	0.1			
2		1.0			0.2	0.8			
3			1.0						0.2
5				0.5	0.2	0.1			
6							0.7	0.3	0.25
7							0.1	0.2	0.1
8							0.1	0.2	0.2
9								0.3	0.25

**Table 3:** Parameters for the fish in dynamical food chain model.

Target tissue	Bone	Flesh	Organs	Stomach
Weight fraction *f*	0.12	0.80	0.05	0.03
Target tissue modifier (TTM)	0.5	1	0.5	0.5
Biological half-life of non-piscivorous fish (d)	500	75	20	3
Biological half-life of piscivorous fish (d)	1000	150	40	5
Biological half-life of demersal fish (d)	500	75	20	3
Biological half-life of bottom predator fish (d)	1000	150	40	5
Biological half-life of coastal predator fish (d)	1000	150	40	5

It is well known that the uptake of caesium and strontium decreases with increasing salinity due to the increase in concentration of competing ions of potassium and calcium, respectively. For caesium it was taken into account when introducing the salinity-dependent correction factor *F_K_* for phytoplankton and macroalgae because caesium enters the food web primarily through the lowest trophic level whereas the contribution of direct uptake from water is minor [13]. Instead of using fixed concentration factors, the *BCF* for ^137^Cs is related to potassium concentration via the electrochemical competition for which the parameters are based on laboratory experiments with marine plants. Then the *BCF*s for phytoplankton and macroalgae can be expressed by:

(22) $$\begin{array}{cc} \mathrm{BC}F_{\mathrm{ph}}=F_{K}\mathrm{BC}F_{\mathrm{ph}}^{*}, & \mathrm{BC}F_{\mathrm{ma}}=F_{K}\mathrm{BC}F_{\mathrm{ma}}^{*} \end{array},$$

where $BCF_{ph}^{*}$ = 20 L kg^−1^ and $BCF_{ma}^{*}$ = 50 L kg^−1^ are standard *BCF*s for ^137^Cs in marine environment [12],

(23) $$F_{K}=\frac{0.05}{\exp\;\left(0.73\;\ln\;(K^{+}/39.1)\;-1220/T\right)},$$

*K^+^* is the potassium concentration (mg∙L^−1^) and *T* is temperature (K).The concentration of potassium varies from the typical value for rivers of 0.5 mg L^−1^ till the typical value in the marine environment of 400 mg L^−1^. For water with a *K^+^* concentration of above 1.5 mg L^−1^, the potassium concentration could be linked to the salinity using the following linear relationship:

(24) $$\begin{array}{cc} K^{+}=11.6\cdot S-4.28 & (K^{+}>1.5mgL^{-1},\;\;\;S>0.14gL^{-1}), \end{array}$$

where *S* is the salinity (g L^−1^).

For strontium, the direct gill uptake is more important due to the lack of bioaccumulation through the food web. The gill extraction coefficient *b_i_* is based on empirical correlations derived from measured equilibrium levels in the seas and can be expressed by:

(25) $$b_{i}=0.1{\left(11.16\cdot S+9.5\right)}^{-1.35}$$

According to the review of radiological data [14,15], every radionuclide is mainly accumulated in a specific tissue (target tissue). It can be assumed that the target tissue controls the overall elimination rate of the nuclide (*T*_0.5_) in the organism. The radioactivity in the food for the predator is then the activity concentration in the target tissue diluted by the remaining body mass of the prey fish, calculated by multiplying the predicted level in the target tissue by its weight fraction. To calculate the concentration in the edible part of fish (flesh) from the calculated levels in the target tissues, a target tissue modifier (TTM) is introduced. This is also based on tissue distribution information as reported by [14,15]. Values of described parameters for the dynamic food-chain model are listed in Table 3.

### Sources of activity

The POSEIDON-R model can deal with four types of routine and accidental radioactive releases:

(i) atmospheric deposition directly on the sea surface;
(ii) runoff of land deposited radionuclide;
(iii) point sources associated with routine releases of nuclear facilities, located either directly at the coast or inland at river systems;
(iv) point sources associated with accidental releases located in any box of the model domain.

For coastal discharges, it is useful to provide a more detailed description in the area close to the release point. For this purpose, the additional “coastal” boxes are nested into the large (“regional”) boxes in the box system of the considered region. There are some assumptions and restrictions to the approach. These are as follows: (i) a coastal box has one vertical layer for the water column; (ii) a coastal box interacts with the surface layer of the surrounding regional box only, the depth of a coastal box is therefore less or equal to that of the surface layer of regional box; (iii) the exchange fluxes with the adjacent regional box are equal in both directions, i.e. only lateral diffusion is taken in account; (iv) only one coastal box can be added per regional box; (v) a coastal box contains at most one source of radioactivity. When calculating the radionuclide concentration in fish in small coastal boxes, the random fish migration should be taken into account. Following [7], the right hand side of Eq. (20) for radionuclide concentration in fish, both in the inner and outer compartments, is extended by the term $(C_{fish}^{out}-C_{fish}^{in})/T_{migr}$ for the coastal compartment and by the term $-(C_{fish}^{out}-C_{fish}^{in})/(\delta T_{migr})$, for the outer compartment. Here *T_mig_*_r_ is the characteristic time of fish migration from a coastal compartment, depending on compartment scale and fish species, and δ is the ratio between the volumes of the outer and the coastal compartments.

POSEIDON-R has also the possibility to deal with off-shore point releases (e.g. for evaluation of the impact of sunken vessels, nuclear submarines, and off-shore waste dumping). In that case, it is possible to use a so-called “local” box. The off-site local boxes have the following features: (i) a local box can be placed at any point in the surrounding regional box at any depth; (ii) the volume and thickness of the local box are calculated as proportional parts of the outer regional box; (iii) as in the case of the coastal box, the exchange flows between the local box and the surrounding regional box are assumed to be equal in both directions.

### Numerical solution

The problem is described by a set of ordinary differential equations, which may be written in a vector-matrix notation as:

(26) $$\frac{dC}{dt}=AC+Q_{re},$$

where C is the concentration vector; *A* is the coefficient matrix that includes water fluxes between boxes, parameters of the food-chain model, etc; $Q_{re}$ is the vector for the release term. Step-like variations of the release in time are assumed, and the implicit Matrix Exponential Method [16] is used to solve a set of Eq. (26). A brief description of the method reads as follows:

An *unforced* (homogeneous) matrix equation,

(27) $$\frac{dC}{dt}=AC,$$

has the solution:

(28) $$C(t)=e^{At}C(0).$$

Over a computational time interval $\left(t_{n},t_{n+1}\right)$ this solution can be expressed as

(29) $$C_{n+1}=e^{A\Delta t}C_{n},$$

or, letting τ be a variable representing the step-size,

(30) $$C(t_{n}+\tau )=e^{A\tau }C(t_{n}).$$

The matrix exponential $e^{A\tau }$ is defined operationally by a truncated Taylor series

(31) $$e^{A\tau }=I+A\tau +\frac{{\left(A\tau \right)}^{2}}{2!}+\frac{{\left(A\tau \right)}^{3}}{3!}+...+\frac{{\left(A\tau \right)}^{k}}{k!},$$

where *I* is the identity matrix (diagonal elements = 1, off-diagonal elements = 0) with the same number of rows and columns as *A*.

For a *forced system* (26) the general incremental solution is:

(32) $$C(t_{n}+\tau )=e^{A\tau }C(t_{n})+e^{A(t_{n}+\tau )}\int_{t_{n}}^{t_{n}+\tau }e^{-At}Q(t)dt,$$

whose exact solution in the case where Q is constant over the step-length is:

(33) $$C(t_{n}+\tau )=e^{A\tau }C(t_{n})+(e^{A\tau }-I)A^{-1}Q(t_{n}).$$

This is the basic equation of the method. The symbol $A^{-1}$ is the inverse of matrix *A* (i.e., $A^{-1}A=AA^{-1}=I$).

Along the instant values of the radionuclide concentration there is a need to compute the time integrated concentration for effective committed dose estimation:

(34) $$IC(t_{n}+\tau )=\int_{0}^{t_{n}+\tau }C(t)dt,$$

(35) $$IC(t_{n}+\tau )=\int_{0}^{t_{n}}C(t)dt+\int_{t_{n}}^{t_{n}+\tau }C(t)dt=IC(t_{n})+\int_{t_{n}}^{t_{n}+\tau }C(t)dt.$$

For $t\in \left(t_{n};t_{n}+\tau \right)$

(36) $$C(t)=e^{A(t-t_{n})}C(t_{n})+\left(e^{A(t-t_{n})}-I\right)A^{-1}Q(t_{n}),$$

hence,

(37) $$\begin{array}{l} IC(t_{n}+\tau )=IC(t_{n})+\int_{t_{n}}^{t_{n}+\tau }\left(e^{A(t-t_{n})}C(t_{n})+\left(e^{A(t-t_{n})}-I\right)A^{-1}Q(t_{n})\right)dt=\\ =IC(t_{n})+C(t_{n})\int_{t_{n}}^{t_{n}+\tau }e^{A(t-t_{n})}dt+\int_{t_{n}}^{t_{n}+\tau }\left(e^{A(t-t_{n})}-I\right)A^{-1}Q(t_{n})dt=\\ =IC(t_{n})+{\left(C(t_{n})\left(e^{A(t-t_{n})}A^{-1}\right)\right|}_{t_{n}}^{t_{n}+\tau }+{\left(\left(e^{A(t-t_{n})}A^{-1}-It\right)\right|}_{t_{n}}^{t_{n}+\tau }A^{-1}Q(t_{n})=\\ =IC(t_{n})+C(t_{n})\left(e^{A\tau }-I\right)A^{-1}+\left((e^{A\tau }-I)A^{-1}-I\tau \right)A^{-1}Q(t_{n}). \end{array}$$

Introducing the notation of the matrices:

(38) $$E1(\tau ):=e^{A\tau },$$

(39) $$E2(\tau ):=\left(e^{A\tau }-I\right)A^{-1},$$

(40) $$E3(\tau ):=\left(\left(e^{A\tau }-I\right)A^{-1}-\tau I\right)A^{-1},$$

Eqs. (33) and (37) were rewritten as:

(41) $$\begin{array}{l} C(t_{n}+\tau )=E1(\tau )C(t_{n})+E2(\tau )Q(t_{n}),\\ IC(t_{n}+\tau )=IC(t_{n})+E2(\tau )C(t_{n})+E3(\tau )Q(t_{n}). \end{array}$$

Matrices *E1*, *E2*, *E3* are linked with the recurrence relation:

(42) $$\begin{array}{l} E1(\tau )=E2(\tau )A+I,\\ E2(\tau )=E3(\tau )A+\tau I,\\ E3(\tau )=\tau^{2}\sum_{k=0}^{\infty }\frac{{\left(A\tau \right)}^{k}}{(k+2)!}. \end{array}$$

The series in (42) can be substituted by the sum with requisite precision.

### Dose module

**Fig. 3:**
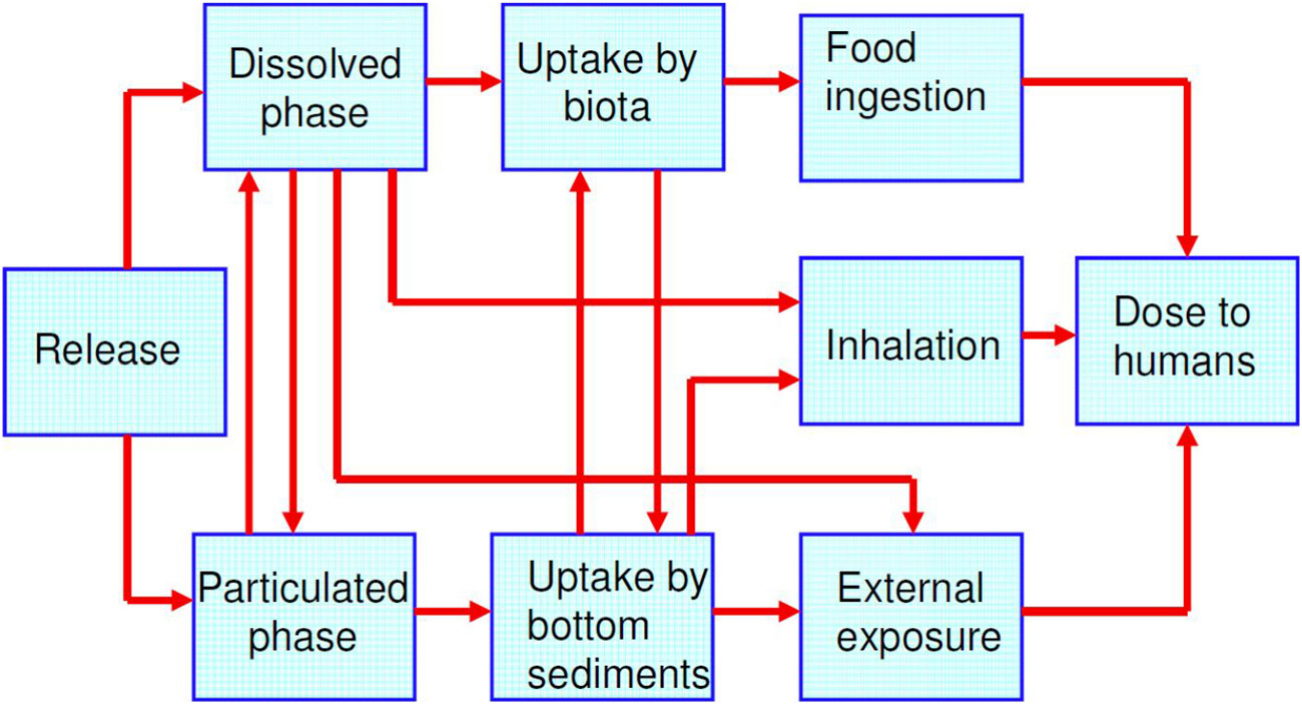
Pathways for human exposure from marine releases of radioactivity (based on [17]).

The POSEIDON-R model includes dose module to assess individual and collective doses to the population due to the regular and accidental releases of radionuclides. The exposure pathways that are considered in the model include: internal exposure through ingestion of seafood and inhalation via sea spray and external exposure through swimming, boating and beach occupancy (Fig. 3).

The annual dose from consumption of marine products *E_marine,k_* (Sv∙yr^−1^) from the ingestion of 8 categories (*f)* of marine products (piscivorous and non-piscivorous fish, demersal, bottom predator, coastal predator, crustaceans, molluscs and macro-algae) for a given box *k* is described as follows:

(43) $$E_{marine,k}=DC_{ing}\left(\sum_{f=1}^{8}C_{f,k}CR_{f,k}\right),$$

where *C_f,k_* (Bq kg^−1^) is the activity concentration of the radionuclide in the marine product of type *f*, *CR_f,k_* is the marine food intake rate (kg y^−1^), *DC_ing_* (Sv Bq^−1^) is the dose coefficient due to ingestion from marine products given by [18].

The annual dose due to inhalation from sea-spray *E_inh,k_* (Sv y^−1^) for a given box *k* is described as follows:

(44) $$E_{inh,k}=DC_{inh}C_{air,k}R_{inh}T_{inh},$$

where *C_air,k_* (Bq m^−3^) is the nuclide specific concentration in air at a given distance from the coast line, calculated from the water concentration using an empirical relationship, *R_inh_* (m^3^ h^-1^) is the inhalation rate for an individual, which is assumed 7300 m^3^ y^-1^. The *DC_inh_* (Sv Bq^-1^) is the dose coefficient for inhalation [18] and *T_inh_* is the occupancy time (h y^-1^).

The external dose from beach material *E_beach,k_* (Sv y^−1^) for a given box *k* is described as follows:

(45) $$E_{beach,k}=DC_{soil}\frac{{\bar{C}}_{1,k}}{60}T_{beach},$$

where ${\bar{C}}_{1,k}$ (Bq m^−2^) is the nuclide specific surface activity concentration in the shore and beach sediment, *DC_soil_* (Sv h^-1^ per Bq kg^-1^) is the dose coefficient for external exposure [18], and 60 is the areal density of the sediment layer (kg m^−2^), *T_beach_* is the occupancy time on the beach (h y^-1^).

The external annual dose from swimming *E_swim,k_* (Sv y^−1^) for a given box *k* is described as follows:

(46) $$E_{swim,k}=DC_{subm}C_{w,k}T_{swim},$$

where *DC_subm_* (Sv h^−1^ per Bq m^-3^) is the dose coefficient for full submerging in water [18], *C_w,k_* is the concentration of radionuclide in the surface water layer (Bq m^-3^) and *T_swim_* is the occupancy time for swimming (h y^−1^).

The external annual dose for boating *E_boat,k_* (Sv y^−1^) for a given box *k* is analogue to the dose for swimming *E_swim_* and is described as follows:

(47) $$E_{boat,k}=0.5\;DC_{subm}C_{w,k}T_{boat},$$

where *T_boat_* is the occupancy time for boating (h y^−1^). As a conservative assumption the dose coefficient is taken as half the dose coefficient for full submerging in water, to consider only exposure from the water surface below the boat.

The total annual dose *E_t_* (Sv y^−1^) is calculated by summing all individual pathways together. The effective committed dose can be calculated by integration in time for a prescribed time period. The doses for non-human biota are calculated using the ERICA Tool (v1.0) methodology [19].

### Case study

**Fig. 4:**
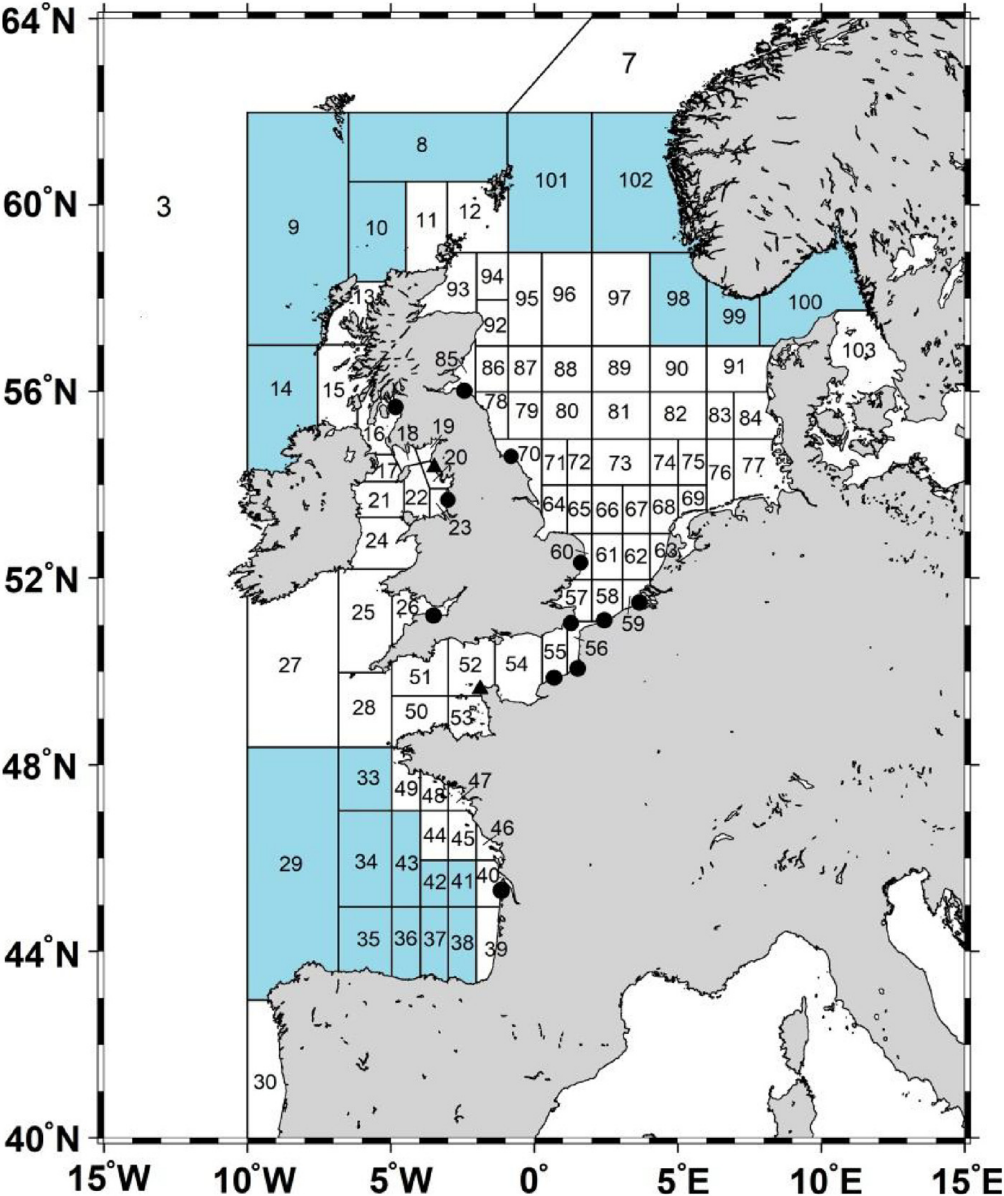
Box system for northeast Atlantic. Black circles denote the active nuclear power plants in the region. Black triangles denote reprocessing plants Sellafield and La Hague. Single-layer boxes are marked in white, while the others are marked with blue.

**Fig. 5:**
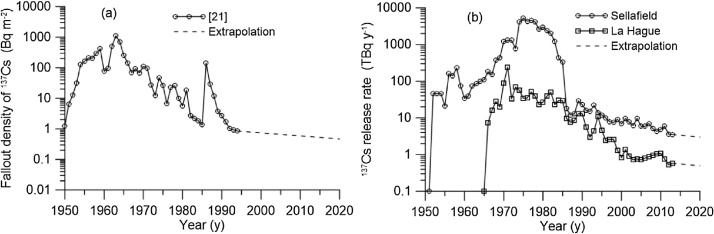
(a) Annual deposition density of ^137^Cs [21]. (b) Release of ^137^Cs from the Sellafield and La Hague reprocessing plants [22].

In the case study we considered transport and fate of ^137^Cs in the northeast Atlantic shelf seas (Fig. 4) released in the period 1950–2020 due to the global deposition after nuclear weapon testing and Chernobyl accident (Fig. 5a) and due to the routine discharges from the Sellafield and La Hague reprocessing plants (Fig. 5b). The box system for the North-Eastern Atlantic includes 108 boxes and covers North Sea, Irish Sea, English Channel, Biscay Bay and adjacent ocean areas. Volume and average depth for each box was calculated using the bathymetry data from [20]. Deep boxes were subdivided on three vertical layers to describe the vertical structure of the radioactivity transport in the upper layer (0–100 m), intermediate layer (100–500 m), and deeper layer (> 500 m). These boxes are marked by blue in the Fig. 4. Water fluxes between boxes were calculated by averaging over 10 years of three-dimensional currents calculated from reanalysis [20]. Notice that the water balance should be checked for each box to satisfy the mass conservation. The particular attention should also be paid to an accurate description of advective and diffusive water fluxes through narrows, such as the Northern Passage in the Irish Sea, which control the transfer of contamination to the open ocean.

The simulation of transport and fate of ^137^Cs in the North-Eastern Atlantic was carried out for the period 1950–2020. The main sources of ^137^Cs as included in this simulation are: (i) global deposition from the weapon testing and from Chernobyl accident [21], (ii) release from the Sellafield and La Hague reprocessing plants [22], (ii) river runoff, and (iv) flow of activity through boundaries. Temporal variations of the sources (a) and (b) are shown in Fig. 5. The flux of ^137^Cs from five main rivers (Elbe, Rhine, Seine, Loire, and Garonne) was estimated by using a generic river runoff model [23]. The flows of ^137^Cs activity through open boundaries of the computational domain were estimated by using observations from the MARiS (Marine Information System) database [24].

**Fig. 6:**
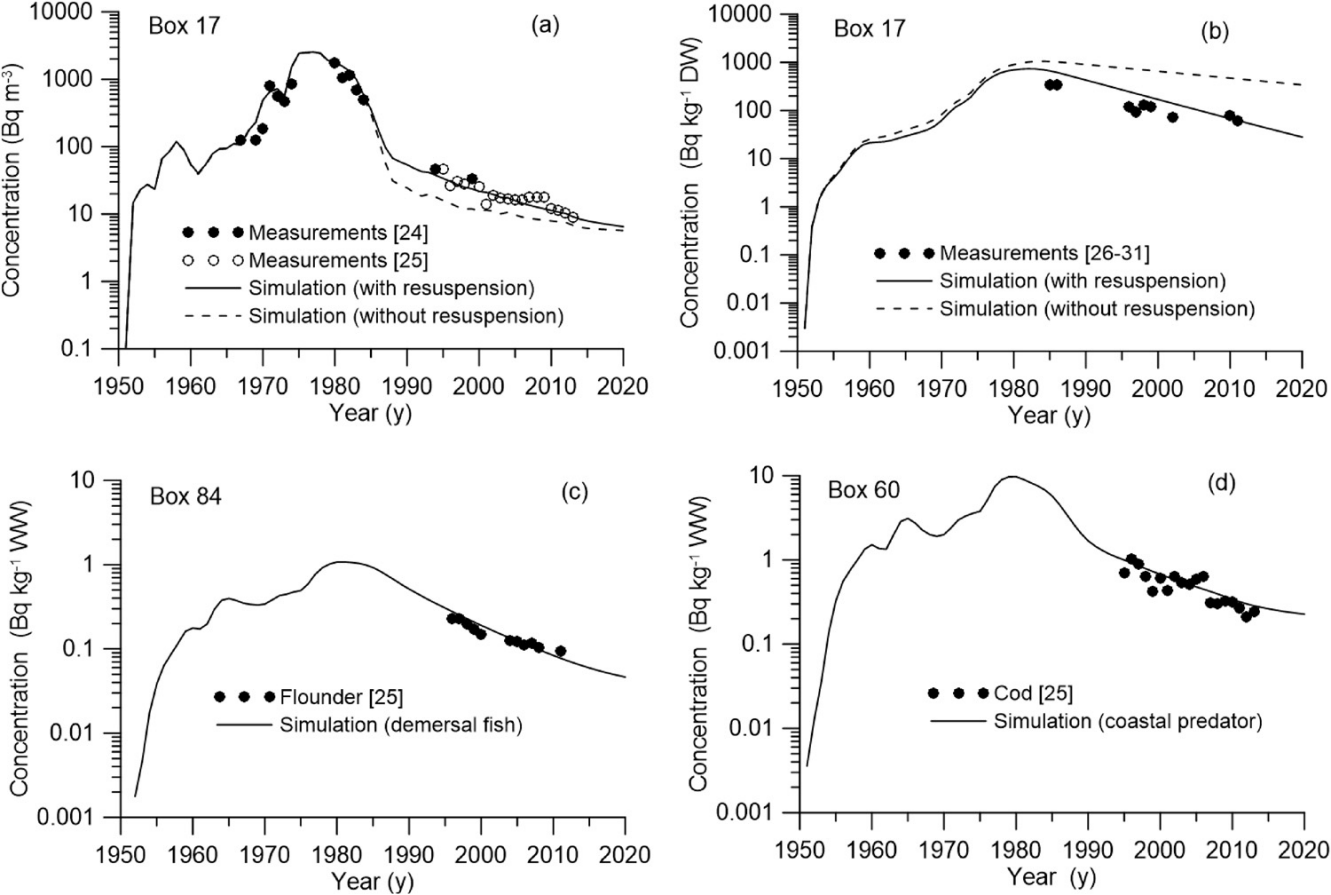
Comparisons between simulated (solid lines) and measured (circles) ^137^Cs concentrations in (a) water, (b) bottom sediments, and (c,d) fish in the boxes. Values in sediment and fish are given in becquerels (Bq) per kilogram (kg) of dry weight (DW) and wet weight (WW), respectively.

Results of simulation for period 1950–2020 are given for different boxes in Fig. 6. As seen in Fig. 6a and b, accounting for resuspension processes has significantly improved the agreement between measurement data and model predictions in the shallow Irish Sea. Measurements in the surface water in the modelling domain were used from the MARiS and OSPAR databases [24,25], whereas measurements of ^137^Cs concentration in the bottom sediments of the Irish Sea were collected from the publications [[26], [27], [28], [29], [30], [31]]. The modelling agrees well with measurements [25] of ^137^Cs concentrations both in the demersal fish (flounder, *Pleuronectes platessa*) and in the coastal predator (cod, *Gadus morhua*) (Fig. 6c,d). Observations also confirm the importance of the activity pathway from organic sediments to the demersal fish, bottom and coastal predators (Fig. 1b). The geometric mean (GM) of the simulated-to-observed ratios for concentration in the water and sediment are 0.99 and 1.4, respectively. The geometric standard deviation (GSD) for concentration in water is 1.85 for a total number of observations *N* = 790 in the whole modelling domain, whereas the corresponding value for concentration in the sediment is of 1.5 for a total number of observations *N* = 29 in the Irish Sea. The GM for the different species of fishes is in the range 1.19–1.27, whereas the corresponding GSD was in the range 1.25–1.34 for the whole modelling domain and *N* in the range 33–67.

The detailed comparison of simulation results and measurements in the Baltic and Black seas and off the Pacific coast of Japan during 1945–2020 due to the weapon testing and accidents at the Chernobyl and Fukushima Dai-ichi nuclear power plants was given in [32]. It was shown that results of simulations conducted with generic parameters agreed well with measurements of ^137^Cs concentrations in the water, bottom sediments, and in fish for the basins with very different marine environment.

## Summary

A detailed description of the three-dimensional compartment model POSEIDON-R for the prediction of transport and fate of radionuclides and their daughter products in the marine environment is given. This includes the equations for transfer of radionuclides in the water and bottom sediment compartments along with a dynamical food chain model for radioactivity transfer in the pelagic and benthic food web. Furthermore, details on the numerical method to solve the model equations are also presented. Novel features of the POSEIDON-R model include: a flexible box system that can handle several types of routine and accidental radioactive releases, “local” boxes to describe off-shore releases and a dynamic food web model which describes both pelagic and benthic pathways of radioactivity transfer in biota. These features are essential for use in emergency response software to nuclear accidents, while models based on a standard box modelling methodology [3] lack such properties. Future development on the dynamic food web model should include effects of fish migration, which till now has been described in the model as a random diffusion process only. Comparison of the model predictions with measurements in the northeast Atlantic seas demonstrates good agreement and confirms detailed model validation [32], which was done for three different marine environments (the Baltic and Black seas, and Japan shelf) using the same generic model parameters. We conclude that the POSEIDON-R is robust software suitable for the decision support systems for emergency response to nuclear accidents (e.g. RODOS [4,5]).

## Conflicts of interest

The authors declare that they have no conflicts of interest.
